# Nonsyndromic Bilateral Multiple Impacted Supernumerary Mandibular Third Molars: A Rare and Unusual Case Report

**DOI:** 10.1155/2013/857147

**Published:** 2013-02-05

**Authors:** G. Siva Prasad Reddy, G. V. Reddy, I. Venkata Krishna, Shravan Kumar Regonda

**Affiliations:** Department of Oral and Maxillofacial Surgery, Panineeya Institute of Dental Sciences, Road No. 5, Kamala Nagar, Dilsukhnagar, Hyderabad, Andhra Pradesh 500060, India

## Abstract

A supernumerary tooth is that which is present additionally to the normal series and can be found in any region of the dental arch. An impacted tooth is defined as the one which is embedded in the alveolus, so that its eruption is prevented, or the tooth is locked in position by bone or the adjacent teeth. The occurrence of multiple supernumerary teeth in only one patient in the absence of an associated systemic condition or syndrome is considered as a rare phenomenon. The occurrence of supernumerary teeth in the lower molar region is rare. A prevalence of less than 2% of cases occurring in this region has been estimated. Their occurrence presents a clinical problem for orthodontists and oral surgeons. The cause, frequency, complications, and surgical operation of impacted teeth are always interesting subjects for study and research. An impacted tooth can result in caries, pulp disease, periapical and periodontal disease, temporomandibular joint disorder, infection of the fascial space, root resorption of the adjacent tooth, and even oral and maxillofacial tumours. The management of impacted wisdom teeth has changed over the past 20 years from removal of nonsymptomatic third molars to simple observation. The aim of this paper is to present a rare case of bilateral multiple impacted supernumerary mandibular third molars.

## 1. Introduction 

Supernumerary teeth may be defined as any teeth or tooth substance in excess of the usual configuration of 20 deciduous and 32 permanent teeth. Supernumerary teeth may occur in single, multiple, unilateral, or bilateral and in one or both the jaws.

The occurrence of multiple supernumerary teeth is a rare phenomenon and is often found in association with syndromes such as cleidocranial dysplasia, Gardners syndrome, or cleft lip and palate [1, 2]. Only a few examples of nonsyndromic multiple supernumerary teeth have been reported in the literature [3]. 

The most common supernumerary teeth, listed in order of frequency, are the maxillary midline supernumeraries, maxillary fourth molars, maxillary paramolars, mandibular premolars, maxillary lateral incisors, mandibular fourth molars, and maxillary premolars. Supernumerary teeth occur in the upper jaw ten times more frequently than in the lower jaw [4]. Multiple impacted teeth may be related to metabolic disorders. In some cases, however, impaction of multiple teeth is not accompanied by a fixed complex of symptoms. Although multiple supernumerary teeth without associated syndromes are rare, their occurrence can create a variety of clinical problems such as crowding, delayed eruption, diastema, rotations, cystic lesions, and resorption of the adjacent teeth. Hence, suitable treatment after proper clinical and radiographic evaluation is essential.

The aim of this case report is to document a rare and an unusual case of multiple impacted supernumerary mandibular third molars in an adult patient which is a unique presentation in the absence of any syndrome and to discuss our proposed clinical approach.

## 2. Case Report

**Figure 1:**
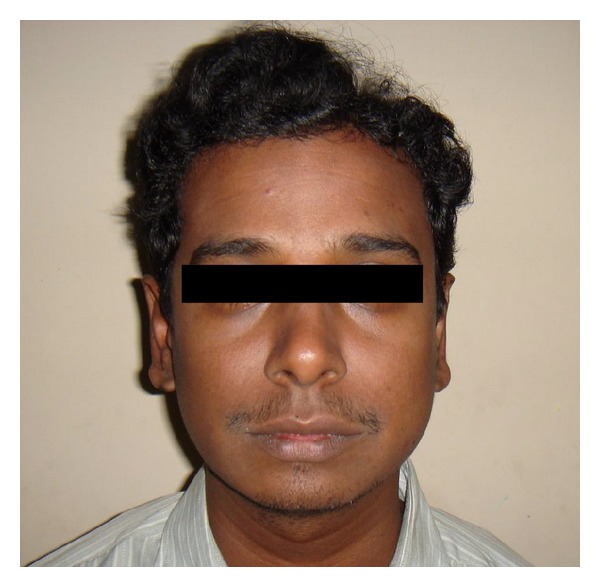
Frontal view of the patient.

A 22-year-old man was referred by a private dental practitioner to our unit with a chief complaint of pain in the lower right back tooth. The medical history, family history, and extraoral examination were not suggestive of any syndrome or metabolic disorder. General physical examination did not show any abnormality (Figure 1). 

**Figure 2:**
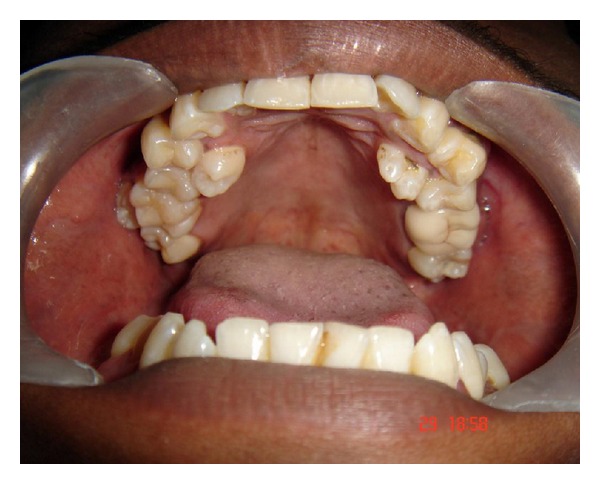
Intraoral photograph showing supernumerary teeth in the maxillary arch.

**Figure 3:**
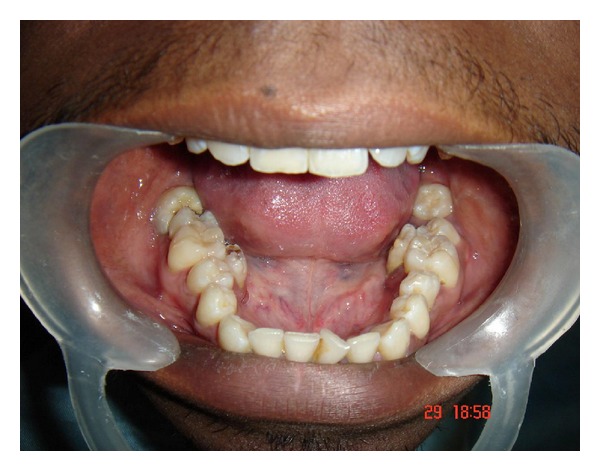
Intraoral photograph showing supernumerary teeth in the mandibular arch.

On local examination the patient had an impacted mandibular third molar which was grossly decayed. Other clinical findings included a total of six supernumerary teeth, four in the maxilla, and two in the mandible (Figures 2 and 3).

## 3. Radiographic Examination

**Figure 4:**
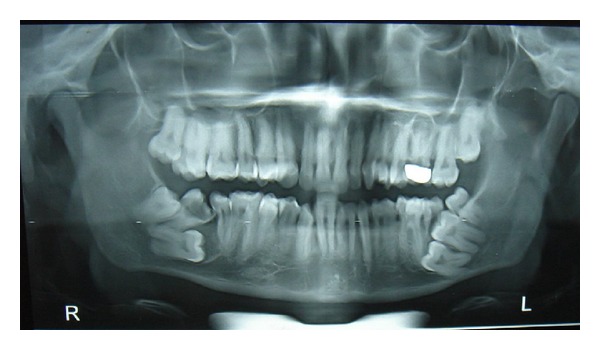
Orthopantomograph showing multiple supernumerary impacted teeth.

In addition to the clinically noticed supernumerary teeth, the panoramic radiograph revealed the presence of impacted multiple supernumerary third molars in the mandible bilaterally (Figure 4). In total, the patient had ten supernumerary teeth. There are six impacted mandibular third molars: three on each side and of which two are supernumerary. 

**Figure 5:**
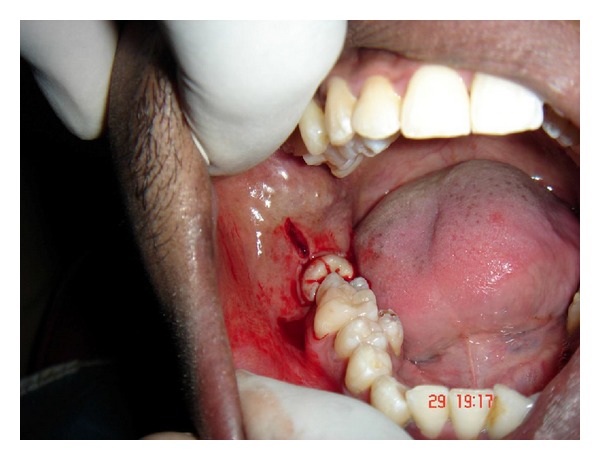
Incision and flap design for removal of mandibular third molar.

**Figure 6:**
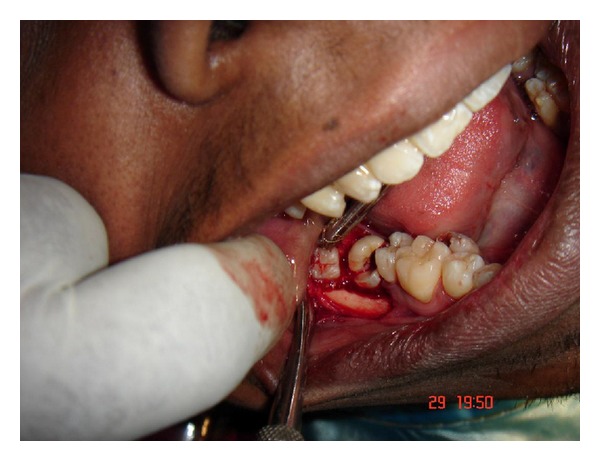
Exposed mandibular impated third molar and supernumerary teeth.

**Figure 7:**
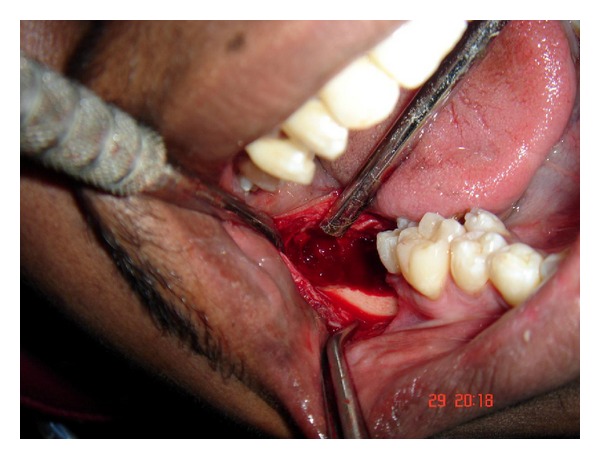
Sockets of extracted mandibular third molar and supernumerary teeth.

**Figure 8:**
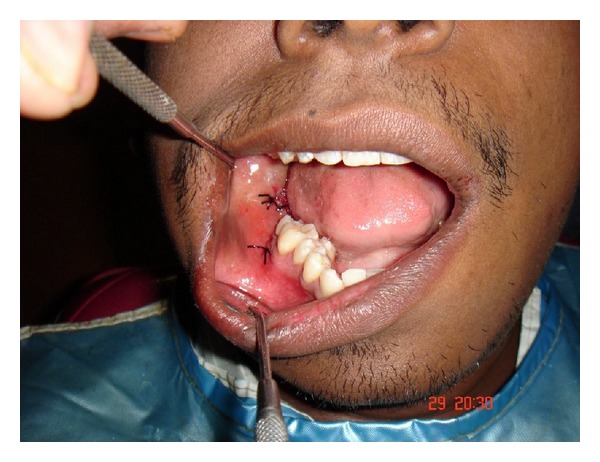
Closure of the surgical wound with mersilk suture.

It was stated that unerupted supernumerary teeth that are asymptomatic do not appear to affect the dentition in any way, and those that are found by chance are sometimes best left in place and kept under observation. In this case, as the chief complaint of the patient was pain on the right side, two impacted third molars were surgically removed on that side under local anesthesia, the third one is left intact as it was asymptomatic, and its removal could cause serious complications like bleeding from the inferior dental canal (IDC) and fracture of the mandible (Figures 5, 6, 7, and 8).

The patient was explained about the surgical removal of the remaining impacted teeth under general anesthesia. He was also explained about the possible complications and their management. The patient did not come for further followup.

## 4. Discussion

The etiology of hyperdontia still remains unclear. Various theories have been proposed to explain the etiology of supernumerary teeth. They are (A) atavism or reversion, (B) heredity, (C) aberrations during embryologic formation, (D) progress zone, and (E) unified etiologic explanation [5]. Hyperactivity of the dental lamina is the most commonly accepted cause. Lack of space or crowding of dental arches, the premature loss of the primary teeth with subsequent partial closure of the area, and rotation of tooth buds are some of the most common causes contributing to impaction.

Supernumerary teeth can be classified according to the following:

- morphology: rudimentary or supplemental;
- number: single or multiple;
- location: mesiodens, paramolars, or distomolars.

The supernumerary teeth present in various different forms. If they are similar to a natural tooth, they are called by the same name, for instance, supernumerary canine, and otherwise, when its morphology is abnormal, it is just indicated as a supernumerary tooth located in a certain area. The occurrence of supernumerary teeth in the region of mandibular molars is less frequent than in the region of maxillary molars. Currently, more than 20 syndromes and developmental anomalies have been associated with supernumerary teeth [6]. The apparently morphologically normal finding of multiple supernumerary teeth in the absence of an associated systemic condition or syndrome is an uncommon phenomenon.

Supernumerary teeth may erupt normally or remain impacted, but in either case their presence may lead to clinical problems. Most problems associated with supernumeraries are because of their potential to interfere with normal occlusal development or with orthodontic mechanics such as crowding, separation, impaction, or delayed eruption of permanent teeth, malocclusion, rotations, retained deciduous teeth, palatally displaced permanent canines, abnormal eruption sequence, and compromised space closure. In addition to these, supernumerary teeth can also cause cyst formation. Hirose et al. [7] reported a case of multiple impacted teeth in upper jaw associated with large follicular cyst in the right maxillary sinus.

Radiographic examination is required to diagnose impacted supernumerary teeth, their position, their relation to the adjacent tooth, and the distance of the impacted permanent tooth to the occlusal plane. Whenever supernumerary teeth are diagnosed, single or multiple, a decision regarding the appropriate management should be made carefully. Surgical removal of the teeth may cause damage to adjacent structures. Cozza et al. [8] emphasized the importance of removing supernumerary teeth to eliminate the cause of a possible delayed eruption of the permanent dentition. Jones et al. stated that the removal of mandibular third molar by sagittal splitting of the mandible allows adequate access, controlled removal of bone, and enables the inferior alveolar nerve to be directly identified and protected [9].

In our opinion, the clinical management of multiple supernumerary teeth poses a great challenge to clinicians. Therefore, it is important to initiate appropriate consultation and an interdisciplinary approach for the treatment.
